# ACP-ADA: A Boosting Method with Data Augmentation for Improved Prediction of Anticancer Peptides

**DOI:** 10.3390/ijms232012194

**Published:** 2022-10-13

**Authors:** Sadik Bhattarai, Kyu-Sik Kim, Hilal Tayara, Kil To Chong

**Affiliations:** 1 Department of Electronics and Information Engineering, Jeonbuk National University, Jeonju 54896, Korea; 2 KMET Business Incubation Center, Room 203, Jeonbuk National University, Jeonju 54896, Korea; 3 School of International Engineering and Science, Jeonbuk National University, Jeonju 54896, Korea; 4 Advanced Electronics and Information Research Center, Jeonbuk National University, Jeonju 54896, Korea

**Keywords:** anticancer peptides, ada-boosting algorithm, data augmentation, binary profile feature, amino acid index, amino acid composition

## Abstract

Cancer is the second-leading cause of death worldwide, and therapeutic peptides that target and destroy cancer cells have received a great deal of interest in recent years. Traditional wet experiments are expensive and inefficient for identifying novel anticancer peptides; therefore, the development of an effective computational approach is essential to recognize ACP candidates before experimental methods are used. In this study, we proposed an Ada-boosting algorithm with the base learner random forest called ACP-ADA, which integrates binary profile feature, amino acid index, and amino acid composition with a 210-dimensional feature space vector to represent the peptides. Training samples in the feature space were augmented to increase the sample size and further improve the performance of the model in the case of insufficient samples. Furthermore, we used five-fold cross-validation to find model parameters, and the cross-validation results showed that ACP-ADA outperforms existing methods for this feature combination with data augmentation in terms of performance metrics. Specifically, ACP-ADA recorded an average accuracy of 86.4% and a Mathew’s correlation coefficient of 74.01% for dataset ACP740 and 90.83% and 81.65% for dataset ACP240; consequently, it can be a very useful tool in drug development and biomedical research.

## 1. Introduction

Cancer is currently the second most common cause of death and a leading cause of morbidity worldwide [1]. Rather than being a single disease, cancer is a heterogeneous set of complex disorders marked by unchecked cell proliferation and the ability to quickly spread or invade other parts of the body [2]. Chemotherapy and radiotherapy are two common conventional cancer treatments that are costly and frequently have negative side effects on healthy cells. Additionally, resistance to the existing anticancer chemotherapeutic medicines can develop in cancer cells [3]. Therefore, new anticancer drugs must be developed regularly to slow cancer cell proliferation. Peptide-based therapy provides significant benefits over other small molecule therapies due to the high selectivity, improved tumor penetration capabilities, and minimal toxicity of peptides under normal physiological settings [4,5].

Anticancer Peptides (ACPs) do not interfere with healthy bodily processes; rather, they provide new therapeutic options. The discovery of ACPs has opened new avenues for cancer treatment. ACPs are made up of 10 to 60 amino acids and feature an amphipathic cationic [6] structure that can interact with the anionic lipid membranes of cancer cells, enabling targeted treatment. Therefore, the discovery of new ACPs is critical for successful clinical applications. Experiments have identified and validated an increasing number of ACPs from protein sequences; however, using the experimental method to identify ACPs is time-consuming, laborious, and costly [1,7]. As a result, computational methods for ACP recognition based on robust composition feature vectors with physicochemical properties using boosting algorithms are urgently required.

Numerous computational techniques in the domain of bioinformatics are utilized to solve various types of issues [8]. In particular, Machine Learning-based methods such as computational methods are used for the identification of ACPs. Based on a support vector machine, Anti-CP was the first computational tool to utilize binary profiles and sequence-based features [9]. Chou’s pseudo-amino acid composition (PseAAC) and a local alignment kernel have been introduced for ACP prediction [10]. A computational model based on the optimization of a 400-dimensional feature vector of dipeptide residue components called g-gap features, representing the order and dipeptide composition of amino acids in peptide sequences was proposed for prediction of ACPs in [11]. An SVM was used to depict ACPs using amino acid composition, average chemical shifts, and reduced amino acid composition [12]. In [13], the authors developed a feature representation learning method using a two-step feature selection method to enhance the prediction of ACPs. In [14], the authors developed a generalized chaos game feature representation method for ACP prediction. The applied ensemble learning model for the identification of ACPs used different features and classifiers, and the classifier output was used as input to the SVM for the prediction of ACPs [14,15]. In [15], the authors proposed a novel computational approach for the accurate identification of ACPs using a deep learning algorithm. The authors of [16] developed a novel method called DRACP, using sequence and chemical characteristics for the identification of ACPs. In [17], the authors proposed a deep learning long short-term memory model (LSTM) called ACP-DL to forecast ACPs using high-efficiency feature representation. AntiCP 2.0, an updated model for the prediction of ACPs using various features and different classes of machine learning classifiers on two datasets-ACP740 and ACP240, has been proposed for the prediction of ACPs [18]. A data augmentation method named ACP-DA, which uses sequential features and a multi-layer perceptron (MLP) classifier to predict ACPs using sequential physiocochemical features, has been proposed as well [19].

The number of the ACPs engaged with the above strategies has not surpassed 1000 cases, which is certainly not a huge number. The prediction performance of this strategy can be further improved if additional ACPs are included [20]. In the proposed method proposed in this paper, we use the concatenated features with data augmentation through a boosting classifier called Adaptive Boosting Classifier (ADA) with a base Random Forest learner, and further improve the performance of the ACP prediction method via machine learning. In this method, the binary profile feature (BPF), amino acid index (AAINDEX), and amino acid composition (AAC), which describe the order and composition of the targets along with their physicochemical properties, are concatenated to represent the peptides; the training set is then augmented in the 210-dimensional feature vector. The augmented training samples are then used to train a machine learning model for ACP prediction.

**Figure 1:**
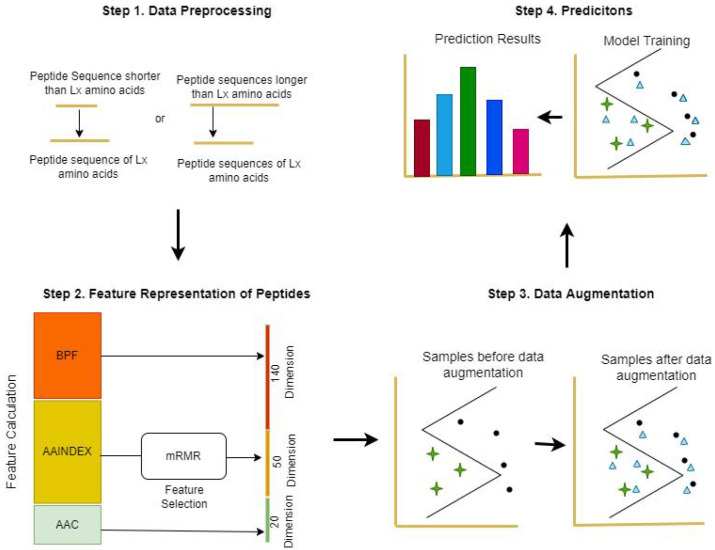
Step flow diagram of ACP-ADA: binary profile feature (BPFs), amino acid index (AAINDEX) features after feature selection, and amino acid composition (AAC) were integrated to represent peptides, and the samples in the training set were augmented in the feature space. After data augmentation, the samples were used to train a machine learning model for the prediction of anticancer peptides (ACPs).

There are four steps involved in the proposed method, as shown in Figure 1. First, the given peptide sequences are input and each peptide sequence is preprocessed to an equal length. Second, we calculate the BPF (140-Dimensional feature vector), AAINDEX (50-Dimensional feature vector with the features selected based on minimum redundancy–maximum relevance (mRMR)), and AAC (20-Dimensional feature vector) of the peptides to contribute a 210-dimensional feature vector. Third, the training samples are augmented based on the contributing feature vector, and the augmented training samples are used to train the boosting classifier. Finally, to test the performance of the proposed technique, we apply five-fold cross-validation to evaluate ACP-ADA based on two benchmark datasets, ACP740 and ACP240. We assess the effectiveness of this strategy using several classification matrices and the outcome of augmentation using a different classifier. The results obtained from the experiment demonstrate that data augmentation based on the concatenated hybrid feature vector, that is, BPFs, AAINDEX, and AAC, can improve the prediction of ACPs with the choice of suitable classifiers using data augmentation. Thus, the proposed ACP-ADA method is suitable for prediction.

## 2. Results

In this section, we illustrate the effects of concatenated features (BPF+AAINDEX+AAC) on the performance of the proposed method when using different classifiers with and without data augmentation. Finally, we compare the proposed method with existing methods using a different classifier.

### 2.1. Parameter Discussion

The parameter affecting the performance of the model is Lx, the peptide length after pre-processing, which was selected as a length of 40, 50, or 60. In the data augmentation stage, N is an additional parameter connected to the number of new positive (negative) samples in the model. Thus, N can be set to 100, 200, or 300 percent of the initial positive (negative) sample number.

The prediction performance of the model established based on different values of the Lx parameter, which is the peptide length, and ’N’, which represents the percentage of augmentation for databases ACP740 and ACP240, is presented in Table 1 and Table 2. MCC is a threshold-independent performance evaluation metric that generates a high score only if the classifier correctly predicts most of the positive and negative data instances. Therefore, we chose the best parameters, namely, Lx = 50 and N = 100% for ACP740 and Lx = 50 and N = 300% for ACP240, according to the maximum MCC value. Because ACP240 has fewer samples than ACP740, the value of N is larger for ACP240 than for ACP740, implying that more pseudo-samples are required for ACP240 than for ACP740. In addition, the performance of the model was evaluated on the ACP214 test dataset. The results for ACP-ADA on the independent test dataset are explained in the Supplementary Materials Section S2, Figure S2, Section S2.1.

### 2.2. Comparison with Different Features Performance

BPF and k-mer sparse matrix have proven to be effective in ACP-DL [17]; here, a physicochemical property feature descriptor called AAINDEX has been introduced as a therapeutic peptide predictor (PPTPP) [21]. AAC features were introduced to identify anticancer peptides through an improved hybrid composition using BPF and Physicochemical properties [22]. BPF, AAINDEX, and AAC are introduced in this methodology to build a model with robust and explainable features. To obtain a more effective feature combination, we used the AdaBoost Classifier with random forest as a base learner to build an ACP prediction model and evaluate the feature performance of each model based on the three features and their pairwise concatenation both with and without data augmentation in different peptide models, then chose the best performing classifier as the anticancer peptide predictor [23,24].

**Figure 2:**
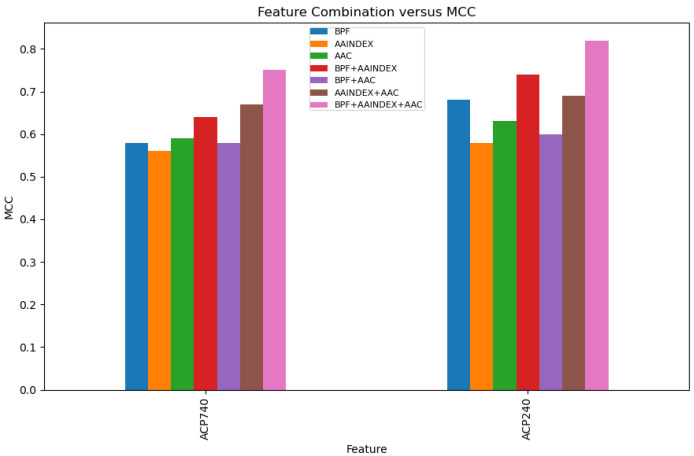
Comparison of feature efficacy for prediction using the BPF, AAINDEX, AAC, and their possible concatenations on the ACP740 and ACP240 datasets.

BPFs, AAINDEX, and AAC are the three features. BPF+AAINDEX, BPF+AAC, AAINDEX+AAC, and BPF+AAINDEX+AAC were combined together. The performance of the models for individual features and their concatenation is depicted in Figure 2. When the three features were applied separately, BPF and AAC performed the best. Based on the MCC value, the BPF+AAINDEX+AAC feature combination produced the best results for ACP740 and ACP240 among the four feature concatenations, as shown in Figure 2. We chose the BPF+AAINDEX+AAC concatenation to represent the peptide sequence based on feature concatenation and consequent performance.The feature importance for anticancer peptide prediction is explained in the Supplementary Materials Section S2.

### 2.3. Classifier Discussion

**Figure 3:**
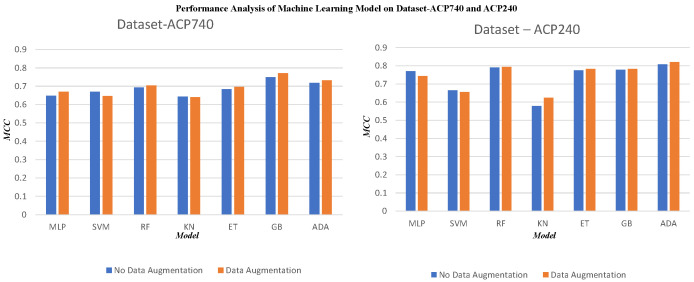
Comparison of the prediction models with and without data augmentation on the ACP740 and ACP240 datasets.

We used the concatenated BPF + AAINDEX + AAC as a concatenated feature to represent peptides. It was then necessary to determine the classifier which worked best with our strategy. In Figure 3, the horizontal axis represents the classifier and the vertical axis represents the MCC value for each classifier with and without data augmentation. We analyzed the performance of the prediction model with and without data augmentation on seven selected models: Multi-layer Perceptron (MLP), a neural network-based model for prediction; Support Vector Machine (SVM), which classifies peptides using a hyperplane; Random Forest (RF), which classifies peptides based on the if–then rule and is a tree-based model; k-Nearest Neighbours (KN), which separates two different classes using their number of nearest neighbours; Extremely Randomized Tree (ET), which is a tree-based hybrid model built using decision trees; Gradient Boosting Classifier (GB), which is a boosting method that focuses on previous incorrect classification by a weak learner and tries to improve the prediction; and AdaBoost (Base Learner = RF), which is an adaptive boosting method constructed using a weak learner random forest. We utilized MCC to assess and test the models’ performance because it is a comprehensive metric. The performance of the selected models on the ACP-740 and ACP-240 datasets are shown in Figure 3.

Figure 3 confirms that based on the ACP740 dataset the prediction models built using MLP, RF, ET, GB, and ADA show performance improvements in terms of the MCC value used to evaluate the prediction models. However, data augmentation causes performance degradation in the models based on SVM and KN. On the ACP240 dataset, data augmentation can enhance the performance of the prediction models developed based on RF, KN, ET, GB, and ADA, meaning that the relative prediction performance of the models based on MLP and SVM decreases. Thus, when using RF, ET, GB, and ADA, data augmentation improved the performance of the ACP prediction model. This finding indicates that the effectiveness of data augmentation is linked to the classifier selected for prediction. Therefore, MLP, SVM, and KN were not suitable for our prediction model.

Based on MCC as the comprehensive metric for evaluating the performance of the model, we chose the AdaBoost classifier (ADA) to build the final predictive model. Though GB the method achieved the best performance on ACP740, its classification performance on the ACP240 dataset after data augmentation, which consists of relatively fewer samples, was much weaker. Therefore, the ADA method was selected as a more robust alternative for classifying ACPs and non-ACPs on both datasets. ADA shows a better performance improvement on both datasets after data augmentation compared to the other classifiers. The method for building the AdaBoost classifier is called ACP-ADA, and has exhibited outstanding performance in various fields in recent years. The results of our developed Adaptive Boosting Classifier for the ACP740 and ACP240 peptide datasets show significant improvement compared with previous state-of-the-art models. It achieves a better performance based on both ACC and MCC, which indicates that the proposed ACP-ADA model can be used as an anti-cancer peptide model for investigating ACPs and non-ACPs.

### 2.4. Comparison with Existing Methods

To ensure the effectiveness and efficiency of the proposed method, we compared the performance of ACP-ADA with ACP-DA [19], ACP-DL [17], AntiCP2.0 [18], and DeepACP [15] while relying on the same main and benchmark datasets and corresponding classification evaluation metrics.

Compared with ACP-DA, the use of our method has a distinct advantage. It is accompanied by a concatenated feature vector (BPF+AAINDEX+AAC) representing the order, composition, and physicochemical properties to represent the peptides with data augmentation and the boosting classifier, which is an ensemble learner that focuses on incorrectly classified samples. The proposed method with concatenated hybrid feature vectors with data augmentation outperforms ACP-DA in most metrics, especially the two most important performance metrics, ACC and MCC.

**Figure 4:**
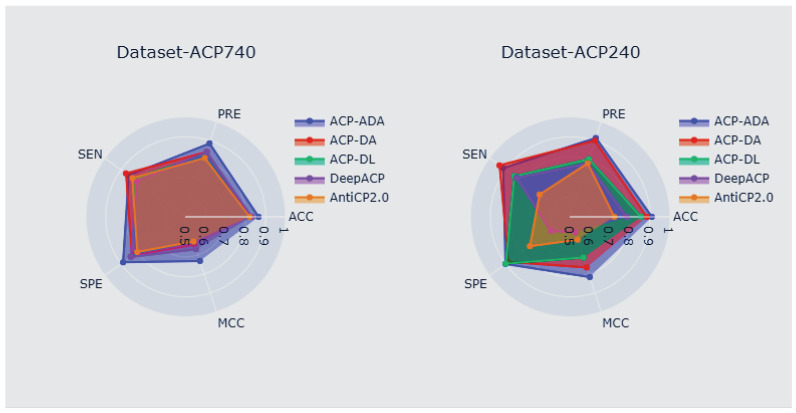
Comparison of ACP-ADA with existing methods on the ACP740 and ACP240 datasets.

As shown in Figure 4, the performance of the proposed method on the ACP740 and ACP240 datasets was better than that of ACP-DA, ACP-DL, DeepACP, and AntiCP 2.0. Compared to the ACP-DA as the current guarding model, our method showed improvements in ACC by 5%, PRE by 5%, SPE by 6%, and MCC by 9% for the ACP740 dataset. For ACP240, the number of samples was lower than for ACP740; nonetheless, our method improved the ACC by 3%, PRE by 1%, SPE by 2%, and MCC by almost 6%. The proposed method outperformed the alternatives on the ACP240 dataset in terms of both the ACC and MCC evaluation metrics, indicating that our strategy is well suited to datasets with a lower fraction of samples. This method applies the Gaussian noise oversampling method with the AdaBoost classifier method using random forest as a base learner and a feature vector representing the order and composition with physicochemical properties, which improves the prediction of ACPs. In addition, the performance of ACP-ADA and all control methods was evaluated on the ACP214 test dataset. The details are provided in the Supplementary Materials Section S2.

## 3. Discussion

Tracing the etiology of cancer remains challenging because of its ambiguous mechanisms. According to a systematic examination, individual feature vectors do not offer viable biomarkers for predicting peptide activity. Therefore, in order to investigate a suitable feature vector, we used BPF, AAINDEX, AAC, and their combination to represent the order, composition, and physicochemical properties of peptides to obtain suitable feature representation. From the experiment with features comparison based on the maximum MCC value for the ACP740 and ACP240 datasets, we selected the concatenation of BPF, AAINDEX, and AAC to represent the peptides. We extracted 210-dimensional feature vectors from this feature combination to represent peptides in the feature space. Here, we propose an ACP prediction method called ACP-ADA which uses a boosting method along with data augmentation of the training samples. According to the results on the two datasets, the proposed model has good overall performance. Compared with existing methods, ACP-ADA had better results in classifying whether the peptides were ACP or non-ACP; its ACC may be attributed to the following reasons.

First, we used effective feature representation methods to characterize peptide sequences. To find the feature combinations, we concatenated three feature representation methods to form robust features using BPF, AAINDEX, and AAC. Experiments on the ACP740 and ACP240 datasets show that the concatenated features obtain the best performance; therefore, we used triad feature combination to represent the peptide sequences.

Second, to compensate for the lack of samples in the training set, data augmentation was applied to generate pseudosamples. We generated a pseudosample by adding perturbation to the training samples in the 210-dimension feature space of the original samples. The feature space of the samples was formed by the concatenation of BPF, AAINDEX, and AAC as a hybrid feature, resulting in a 210-dimensional numerical feature vector. BPF is composed of vectors of 1 and 0, which are incompatible with the addition of noise; thus, we only added noise to AAINDEX and AAC to generate pseudosamples. Augmented training samples were used to train the machine learning model to further improve the performance of the prediction model, which showed a significant impact based on the choice of the classifier.

Finally, the various models showed good performance in many bioinformatic classifications. However, it remains unclear whether data augmentation can improve the performance of prediction models using different classifiers. Therefore, we analyzed the effect of this methodology using seven different classifiers. The results show that data augmentation is effective when using RF, ET, GB, and ADA classifiers with RF as the base learner. Therefore, we selected ADA, which is a boosting classifier, as the final classifier with the best overall performance.

In summary, the proposed method for the identification of ACPs showed improved performance; it is our hope that ACP-ADA can play an important role in biomedical research and the development of new anticancer drugs. Furthermore, a comparative analysis with other methods showed that ACP-ADA was better than the other methods in most cases.

To accurately and quickly identify ACPs, a boosting classifier was applied to discriminate peptide sequences using a 210-dimension feature vector which focuses on incorrectly classified samples as a sample of priority while constructing a random forest to form a complete AdaBoost classifier. As an ensemble learning method, boosting effectively prevents over fitting; it performed well on test data and achieved a comparative improvement in prediction of ACPs. In addition, the secondary and tertiary structure prediction characteristics of peptides can be added to this model as a feature descriptor, which may improve the performance of the model with the data augmentation method. Furthermore, the neural network method can be used for the identification of ACPs with an increase in the dataset size.

Because of the successful result with data augmentation for the dataset with low sample proportion (ACP240 dataset), using machine learning boosting methods, we can conclude that this methodology for peptide data augmentation can be applied for training deep learning models such as Convolutional Neural Networks, Recurrent Neural Networks, Transformer and several language models. Based on our predictive performance improvement for the dataset with a lower number of positive and negative classes, we can assert that this method of peptide data augmentation can enhance and quantify predictive performance on datasets with fewer samples using advanced deep learning models, which can be further explored for peptide-based research using data augmentation to escalate model performance. This method can be explored while working with advanced deep learning models using data augmentation.

## 4. Materials and Methods

### 4.1. Data Acquisition

In this study, a machine learning model called the boosting method is proposed to predict ACPs. Called ACP-ADA, the proposed method uses concatenated features provided by BPF, AAINDEX, and AAC. We evaluated the predictive performance of ACP-ADA for ACPs on the ACP740 and ACP240 benchmark datasets. Furthermore, using the common tool CD-HIT [20], sequences with a similarity of more than 90 percent were eliminated [20,25]; we used similar configuration as previous works for fair comparison on the two benchmark datasets. Therefore, there was no duplicate sequence between datasets, and both were unique and non-redundant. These datasets can be publicly accessed through the https://github.com/haichengyi/ACP-DL (accessed on 24 September 2021, Korea) ACP-DL Dataset Repository.

The main dataset, ACP740, includes 364 non-ACPs (negative examples) and 376 experimentally validated ACPs (positive examples).

The alternate dataset, ACP240, includes 111 non-ACPs (negative examples) and 129 experimentally validated ACPs (positive examples).

In addition, we build datasets with an CD-HIT cutoff of 0.35% named ACP614 and ACP214. A description of the datasets and experimental results are provided in the Supplementary Materials Section S2.

### 4.2. Preprocessing

**Figure 5:**
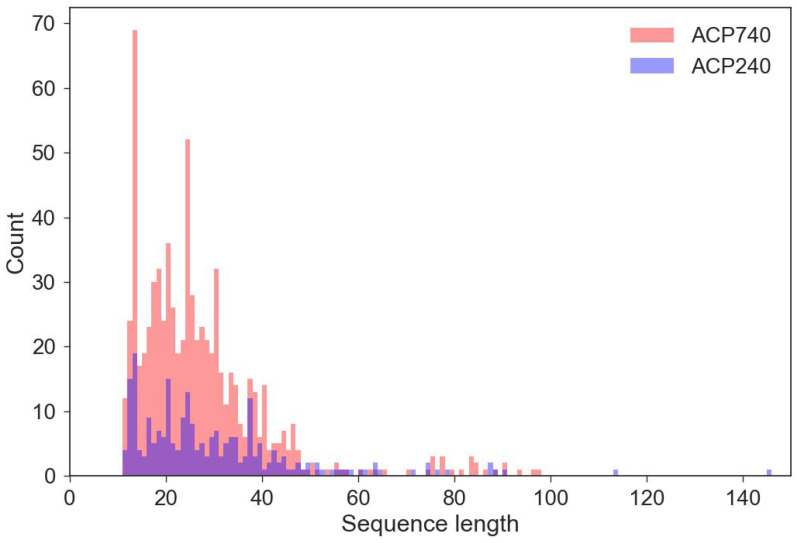
Histogram of the sequence length of peptides in ACP740 and ACP240 datasets.

The iLearn python package [26] can encode peptides of the same length. The lengths of the peptides in the ACP740 and ACP240 datasets were statistically analyzed in order to establish the optimal sequence lengths, which we then used to preprocess the original peptide sequences. As shown in Figure 5, the majority of the peptides were less than 60 amino acids in length. To retrieve peptides of the same length, each peptide was processed as follows. For sequences shorter than Lx amino acids, each peptide was padded with “X” until Lx amino acids were reached. For sequences longer than Lx amino acids, the extra amino acids after Lx were removed; only the first Lx amino acids were retained. Lx was set to 40, 50, or 60 [12,19]. We believe that the best length to represent the peptides can be derived from a peptide length of 40, 50, or 60 for the calculation of BPF, AAINDEX, and AAC.

### 4.3. Feature Extraction

First, the physiochemical characteristics of each sequence of amino acids were determined using the AAINDEX function in the iLearn Python package [27]. Because AAINDEX results in larger dimensional features, mRMR was then used for feature selection. Similar to AAINDEX, the AAC feature descriptors in iLearn Python package were used to calculate AAC features for the entire peptide sequences [28,29]. The BPFs, AAINDEX, and AAC for each sequence were concatenated to represent the order, physicochemical characteristics, and composition of the peptides. The integrated feature for the prediction can be represented as

(1) Feature=BPF+AAINDEX+AAC

BPF represents the residue order, AAINDEX represents the peptides in terms of the properties of 20 amino acid residues with respect to the physicochemical properties (activity-based features) and AAC represents the proportion of residues dominant in ACPs and non-ACPs (which are highly dominant). Thus, the combination collectively represents the residue order, activity, and percentage of each residue for each peptide. Combining these features can capture the local residue level order information, structural sequence features, and proportion of amino acids highly available in ACPs and Non-ACPs as explainable parameters for the sequence and model. Because of this, we selected and extracted BPF, AAINDEX, and AAC as a predicting feature in our proposed method. Each individual feature was used along with the combination of trait features as predictors for the machine learning model. Finally, the training samples were augmented in the feature vector and used to train the machine learning model, with the trained model assigning the class level to the test sets.

The newly constructed datasets ACP614 and ACP214 (with CD-HIT 0.35%) were featured based on PSSM. The details are explained in the Supplementary Materials Section S2.

### 4.4. Representation of Peptides

Converting peptides of various lengths into feature vectors of a fixed length is the primary goal of feature representation. The unprocessed peptide sequence P can be modeled as

(2) P=P[1]P[2]P[3]P[4]…P[L]

where P[1], P[2], P[3], and P[L] in Equation (2) represent the first, second, third and terminating residue peptide of length ’L’, respectively. To train the machine learning model, residue P[i] served as a general representation of amino acids in peptides and a component of the standard amino acid alphabet. The primary step was converting the variable-length peptides into a fixed length in order to calculate the binary profile feature, amino acid index, and amino acid composition to represent the peptide sequences. In this study, we introduced three feature representation methods through the concatenation of BPF and AAC with physicochemical properties called the AAINDEX, as described below; the peptides can be expressed in terms of a fixed length ‘Lx’ for sequences, formulated in Equation (3) as follows:

(3) P=P[1]P[2]P[3]P[4]…P[Lx]

#### 4.4.1. BPF

The binary profile has the advantage of providing an order of residues in the peptides, which is not feasible with composition-based characteristics [30,31]. As a result, binary profile traits can distinguish peptides that are chemically similar and functionally distinct. It was difficult to build a fixed-length pattern because the lengths of the peptides employed in this investigation were different. To solve this problem and generate a fixed-length pattern, we isolated fixed-length segments from the N-terminus to represent the peptide, with each amino acid type represented using a 0/1 feature vector. The first type of amino acid in the alphabet was encoded as f(A) = 1,0,0,0...,0), whereas the second type of amino acid was encoded as f(C) = (0,1,0,0...,0). The N-terminus of a particular peptide sequence P with a length of k amino acids was encoded as the feature vector represented in Equation (4), expressed as follows:

(4) F(BPF[k])=[f(P[1],f(P[2],…f(P[k])]

where k represents the length of the peptide resembling the N-terminal amino group. The experiments suggest that setting k to 7 produces the best results [17,19]. As a result, the BPF vector encoded a particular peptide sequence into a 20 × 7 feature vector.

#### 4.4.2. AAINDEX

The most useful qualities for representing biological reactions are the physicochemical characteristics of amino acids, which have been widely employed in bioinformatic studies. Numerous published indices that represent the physicochemical characteristics of amino acids can be found in the AAINDEX database [4,10,30], including a set of 20 numerical values for each physicochemical property for all amino acids. The AAINDEX database’s 544 physicochemical attributes were retrieved, returning a total of 531 physicochemical characteristics to represent each residue in the peptide sequence; any physicochemical qualities for any of the amino acids that were removed are indicated with “NA”. The AAINDEX descriptor can be used to encode peptides of the same length [32]. When Lx is set to 40, the AAINDEX descriptor for a peptide of length 40 produces a feature vector with a dimension of 21,240, which is excessively high and results in a dimension disaster. We chose the best 50 feature vectors to represent the peptide sequences and reduce dimensionality issues using the mRMR approach after the physicochemical properties of peptides (AAINDEX) were extracted using the iLearn platform.

#### 4.4.3. AAC

The frequency of each residue in the peptide sequence was determined using AAC encoding. AAC, which demonstrates that particular residues are more prevalent in ACPs than in non-ACPs, can be used to discriminate between ACPs and non-ACPs. As a result, the AAC feature was added to represent the peptide, then extracted into a fixed-dimensional feature vector using the iLearn Python tool. All 20 natural amino acid frequencies (i.e., “ACDEFGHIKLMNPQRSTVWY”) can be described by Equation (5):

(5) $$F(\mathrm{AAC}[N])=\frac{N(t)}{N},t\in \left(A,C,D,\ldots Y\right)$$

Here, N(t) is the repetition of an amino acid of type t, N is the length of a protein or peptide sequence, and F(AAC[N]) results in a 20-dimensional feature vector representing the AAC of the peptide sequence. A conjoint feature vector was formed to represent peptides using BPF (140), Amino AAINDEX (50), and AAC (20); the new feature vector dimension was 140 + 50 + 20 = 210-dimensional feature vector. In addition to sequential order information features and sequential composition features, we calculated PSSM features for the newly constructed datasets; a detailed description is provided in the Supplementary Materials Section S2.

### 4.5. Data Augmentation

When solving scientific problems, data imbalance and insufficient data are common issues in machine learning and deep learning technologies [30]. Historically, data augmentation been employed in the field of computer vision to handle this challenge, which can involve flipping, scaling, zooming, translating, and cropping the original sample [13,18]. Data augmentation can help to solve data imbalance issues. Here, the problem of a small sample size can be fixed by enhancing the data. Techniques for noise-added oversampling, which produce faux samples by perturbing the original samples in the feature space, can be used to create new samples. To enhance the effectiveness of the ACP prediction model, the number of positive and negative samples in the datasets was increased using peptide data augmentation techniques. The characteristics of the peptides were divided into three sections, namely, BPFs, AAINDEX, and AAC. BPFs are binary codes consisting of 0 and 1, and as such are not suitable for adding perturbations, as adding a noise value to the bits results in loss of the order information. Only the AAINDEX and AAC are susceptible to perturbation. The mathematical method for generating new samples F(new) for training the model is mathematically described by Equation (6):

(6) F(new)=F(i)∗V∗a+F(i)

where F(i) is a random sample from a training sample of peptide sequences, i = 1 …, and N (N) is the total number of positive (negative) samples, representing a 210-dimensional vector used to generate a perturbation that corresponds to F(i). In order to improve model learning, we performed peptide augmentation by adding noise to the training samples following the Gaussian distribution and left the test set without data augmentation. Because test sets are used for evaluation of model performance, they are not suitable for data augmentation. Here, V is composed of three parts; one is a 140-dimensional vector of zeros and ones corresponding to BPFs, and the other consists of a 50-dimensional random vector and a 20-dimensional random vector with a value between 0 and 1, corresponding to the AAINDEX and AAC, respectively. Thus, perturbation was added to AAINDEX and AAC and BPFs were kept unchanged in the pseudo-sample set F (new), where ‘a’ is the coefficient of perturbation and was set to 0.02 for ACP740 and 0.005 for ACP240.

We tried adding different values of perturbation, and usually preferred a range of 0 to 1 to ensure that the features followed a Gaussian distribution. After training and testing with different set values, we found 0.02 and 0.005 to be the best values to add for feature distribution for ACP740 and ACP240, respectively, as these values closely resemble the AAINDEX and AAC. Augmenting the samples with these values led to improved prediction performance. Therefore, these fixed values of noise were considered as standard for augmenting the samples in ACP740 and ACP240. To obtain N new samples, the sampling process was repeated N times using these noise value for datasets ACP740 and ACP240.

### 4.6. Classifier for Prediction

AdaBoost Random Forest Model

When adaptive boosting is used in conjunction with the random forest approach, there are two options. The first is “boost in the forest”, in which an AdaBoost classifier is generated for each random vector k (i.e., a set of variables); a series of ‘simple’ AdaBoost classifiers, each with a limited number of variables, is then used to arrive at a final result [33]. Here, we instead use a different approach in which a random forest is used as a poor learner. It is clear from a numerical standpoint that AdaBoost works faster with simple weak learner algorithms than with forests with trees, which is important for real-time applications; the philosophical idea behind weak learner algorithms is to find weak assumptions quickly with a moderate error rate [34,35].

An AdaBoost classifier is a meta-estimator that starts with the original dataset and then fits new copies on the same dataset while adjusting the weights of poorly classified instances in order to ensure that succeeding classifiers focus on more difficult cases. Owing to its excellent performance, this classifier has gained popularity in many fields of bioinformatics [36,37]. To build the model, we used the scikit-learn Python package; we developed the AdaBoost model with a random state = 121, number of estimators = 406, and learning rate = 0.04; the other parameters were set to the default values shown in Table 3. This model introduces a parameter for the base learner (random state = 120, number of estimators = 300, minimum number of data points placed in the node before the node is split = 10, minimum number of data points allowed in a leaf node = 1, maximum number of features considered for splitting a node = auto, method for sampling data points(bootstrap) = False), which were identified as the best parameters for the model using five-fold cross-validation. In addition, we evaluated the performance of other classifiers, including MLP (Multi-Layer Perceptron), SVM (Support Vector Machine), RF (Random Forest), KN (k-Nearest Neighbors), ET (Extremely Randomized Tree), GB (Gradient Boosting Classifier), and ADA (Ada Boosting Classifier with base learner Random Forest) to build a prediction model based on the non-augmented data and augmented data in the training set. Among these classifiers, the ADA classifier works best according to the experimental results obtained from the comparison with the features and with and without data augmentation.

### 4.7. Evaluation Metrics of the Model

To evaluate the performance of ACP-ADA, we used a five-fold cross-validation strategy. Five performance metrics were used to evaluate the strength of the binary classification tasks: accuracy (ACC), precision (PRE), sensitivity (SEN), specificity (SPE), and the Mathews correlation coefficient (MCC) [21,22,23,24]. Mathematically, these metrics can be computed as follows:

(7) $$\mathrm{ACC}=\frac{(\mathrm{TP}\;+\;\mathrm{TN})}{(\mathrm{TP}\;+\;\mathrm{TN}\;+\;\mathrm{FP}\;+\;\mathrm{FN})}$$

(8) $$\mathrm{PRE}=\frac{\mathrm{TP}}{\mathrm{TP}\;+\;\mathrm{FP}}$$

(9) $$\mathrm{SEN}=\frac{\mathrm{TP}}{(\mathrm{TP}\;+\;\mathrm{FN})}$$

(10) $$\mathrm{SPE}=\frac{\mathrm{TN}}{(\mathrm{TN}\;+\;\mathrm{FP})}$$

(11) $$\mathrm{MCC}=\frac{\left(\mathrm{TP}*\mathrm{TN}\right)-\left(\mathrm{FP}*\mathrm{TN}\right)}{\sqrt{\left((\mathrm{TP}+\mathrm{FN})(\mathrm{TP}+\mathrm{FP})(\mathrm{TN}+\mathrm{FP})(\mathrm{TN}+\mathrm{FN})\right)}}$$

where FP stands for false positive predictions, FN stands for false negative predictions, TP stands for correct positive predictions, and TN stands for true negative predictions. In addition to these metrics, we used the F1-Score to evaluate the performance of the classifiers. The detailed results are provided in the Supplementary Materials Section S2.

## 5. Conclusions

The proposed ACP-ADA method can be used to determine whether peptides are anticancer or non-anticancer based solely on the concatenation of hybrid sequence feature vectors representing the order, composition, and physicochemical properties with data augmentation. The predicted results obtained by ACP-ADA via five-fold cross-validation on the benchmark datasets ACP-740 and ACP-240 indicate that the proposed ACP-ADA method is comparably better, or at the very least capable of supplementing futuristic computational models in this area. Because of its success rate on the alternate ACP-240 dataset with a lower number of (positive/negative) samples, ACP-ADA is expected to become a useful throughput tool that is widely used in drug development and biomedical research. This confirms the data augmentation method as an alternative approach to over-sampling techniques, as it can boost the performance of various sequence-based peptide and non-peptide models based on the choice of features and classifier. In the future, we intend to consider more complex feature extraction methods and machine learning algorithms to further improve the performance of ACP peptide prediction models.

## Figures and Tables

**Table 1:** Performance of ACP-ADA with parameters ‘Lx’ and ‘N’ on the ACP740 dataset along with performance metrics (the best metrics are shown in bold).

Lx	N%	ACC%	PRE%	SEN%	SPE%	MCC%
40	100	85.81	87.60	84.05	87.64	71.77
40	200	86.21	88.13	84.32	88.17	72.64
40	300	85.54	87.55	83.52	87.64	71.25
**50**	**100**	**86.48**	**88.63**	**84.32**	**88.73**	**73.19**
50	200	85.81	87.82	83.79	87.91	71.81
50	300	85.67	87.83	83.52	87.91	71.58
60	100	86.48	88.61	84.32	88.73	73.05
60	200	85.94	87.84	84.05	87.91	72.05
60	300	86.35	88.33	84.32	88.46	72.86

**Table 2:** Performance of ACP-ADA with parameters ‘Lx’ and ‘N’ on the ACP240 dataset along with performance metrics (the best metrics are shown in bold).

Lx	N%	ACC%	PRE%	SEN%	SPE%	MCC%
40	100	87.08	88.29	87.60	86.44	74.06
40	200	87.09	88.15	87.63	86.48	74.21
40	300	86.66	87.15	88.36	84.66	73.19
50	100	88.33	89.18	89.13	87.35	76.57
50	200	88.34	89.28	89.14	87.39	76.81
**50**	**300**	**90.83**	**91.46**	**91.50**	**90.07**	**81.65**
60	100	90.41	90.78	91.47	89.16	80.75
60	200	89.16	90.51	89.16	89.16	78.30
60	300	90.02	90.67	90.70	89.16	79.91

**Table 3:** Model Parameters for AdaBoost-Random Forest for ACP Prediction.

Parameters	Settings
Base Learner	Random Forest
Learning Rate	0.04
Seed	121
Number of Estimators	406

## Data Availability

The datasets and codes are available at https://github.com/Sadik90/ACP-Prediction/find/main (accessed on 6 July 2022).

## Supplementary Materials

The following supporting information can be downloaded at: https://www.mdpi.com/article/10.3390/ijms1010000/s1. References are cited in [38,39,40,41,42].

## Abbreviations

The following abbreviations are used in this manuscript:

ACP	Anticancer Peptide
BPF	Binary Profile Feature
AAINDEX	Amino Acid Index
AAC	Amino Acid Composition
mRMR	Minimum Redundancy–Maximum Relevance
MLP	MultiLayer Perceptron
SVM	Support Vector Machine
RF	Random Forest
KN	k-Nearest Neighbours
ET	Extremely Randomized Tree
GB	Gradient Boosting
ADA	Adaptive Boosting with Random Forest
