# Supplementary material for: ACP-ADA: A Boosting Method with Data Augmentation for Improved Prediction of Anticancer Peptides
Source: Int J Mol Sci. 2022 Oct 13;23(20):12194. doi: 10.3390/ijms232012194 (PMC9603247; doi:10.3390/ijms232012194)
Supplement: Supplementary file 1 [file ijms-23-12194-s001.zip › Supplementary Material.pdf]

Article

ACP-ADA: A Boosting Method with Data Augmentation for Improved Prediction of Anticancer Peptides

Sadik Bhattarai [1], Kyu-Sik Kim [2], Hilal Tayara [3,*] and Kil To Chong [1,4,*]

1 Department of Electronics and Information Engineering, Jeonbuk National University, Jeonju 54896, Korea
2 KMET Business Incubation Center, Room 203, Jeonbuk National University, Jeonju 54896, Korea
3 School of International Engineering and Science, Jeonbuk National University, Jeonju 54896, Korea
4 Advanced Electronics and Information Research Center, Jeonbuk National University, Jeonju 54896, Korea
* Correspondence: hilaltayara@jbnu.ac.kr (H.T.); kitchong@jbnu.ac.kr (K.T.C.)

Citation: Bhattarai, S.; Kim, K.-S.; Tayara, H.; Chong, K.T. ACP-ADA: A Boosting Method with Data Augmentation for Improved Prediction of Anticancer Peptides. *Journal Not Specified* **2022**, *23*, 12194. https://doi.org/10.3390/ijms232012194

Academic Editors: Serena Martini and Davide Tagliazucchi

Received: 29 August 2022
Accepted: 11 October 2022
Published: 13 October 2022

Publisher's Note: MDPI stays neutral with regard to jurisdictional claims in published maps and institutional affiliations.

Copyright: © 2022 by the authors. Submitted to *Journal Not Specified* for possible open access publication under the terms and conditions of the Creative Commons Attribution (CC BY) license (https://creativecommons.org/licenses/by/4.0/).

1. Problem of Over fitting in Deep Learning Architecture

Deep Learning models like Dense Neural Networks (DNN), Convolutional Neural Networks (CNN), and Recurrent Neural Networks (RNN) are achieving outstanding results in different areas including bioinformatics. However, these models suffer the problem of overfitting if the training datasets are small. We trained 4-Layered DNN, and 4-Layered CNN with a dropout of 0.5 for dataset ACP740, which consists of a large sample proportion compared with ACP240. The result of the training and testing of the models (DNN and CNN) on the dataset ACP740 are shown in Figure S1. These plots show that the trained models overfit after few epochs so they cannot generalized to new data points.

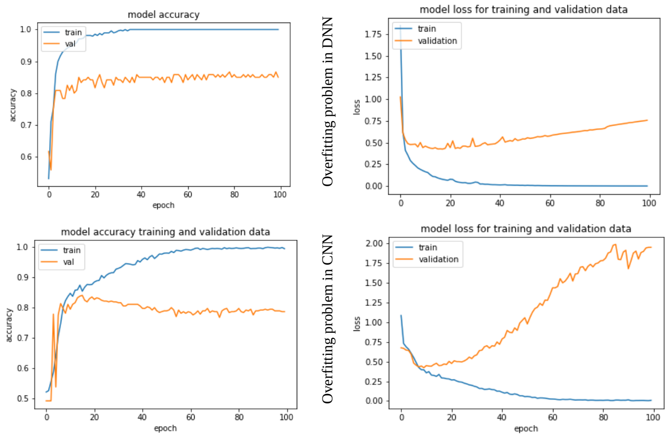

Figure S1. Over fitting Problem in DNN and CNN for Anticancer Peptide Dataset.

2. Dataset construction and featurization

We constructed a new dataset with 0.35% sequence cut-off using CD-HIT program for building new training and testing datasets. The newly constructed training dataset is called ACP614, and the newly constructed test data is called ACP214. ACP214 dataset was used as the independent test data for evaluation of prediction performance of model. ACP614 dataset, consisting of 277 positive sequences labelled as '1' and 337 negative sequences labelled as '0', was used for training the model. ACP214 dataset (independent dataset) integrated the test dataset used in ACP-DL(ACP240),DeepACP(ACP162) and applied CD-HIT of 0.35% for removing high redundancy. It consist of 118 positive sequences labelled as '1' and 96 negative sequences labelled as '0'. Firstly the trained model on dataset ACP740 and ACP240 was evaluated on the independent dataset ACP214 .The result of the model performance is shown in Figure S2.

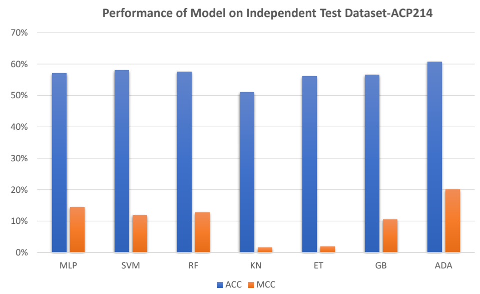

Figure S2. Performance Metrics for Machine Learning Models on independent test dataset ACP214.

The machine learning models shown in Figure S2 were trained on ACP614 using the concatenated feature [BPF+AAINDEX+AAC] with data augmentation. The results show that the adaptive boosting classifier outperformed the other machine learning models. The ACC and MCC of the adaptive boosting method(ADA) with data augmentation on the test dataset ACP214 was 61.56% and 20.04%. Compared to the 5-fold cross-validation method using the data set with 0.90% cut-off, the model performs satisfactorily for the independent dataset ACP214. This result indicated that most of the sequences in the training and test sets were highly redundant in ACP740 and ACP240 and therefore ACP-DA, DeepACP, ACP-DL, and AntiCP2.0 had achieved higher ACC and MCC values.

In addition to the performance of the model, we evaluated the feature importance. Comparing the feature performance on training data sets ACP740 and ACP240, we captured the rank of the features with low priority and high priority. The summary of the feature importance is represented in Figure S3.

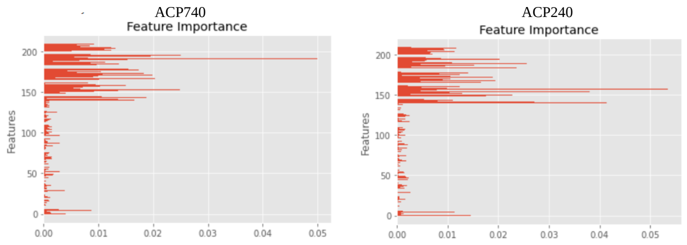

Figure S3. Feature Importance for prediction based on BPF,AAINDEX and AAC.

In Figure S3, the dimension of 0-139 represents the BPF, 140-189 represents the AAINDEX, and 190-209 represents the AAC. AAINDEX has more weight for classifying anticancer peptides whereas the model prioritizes the BPF with a low score for prediction. This justifies that AAINDEX and AAC are good sequential computational features for identifying anticancer peptides in a sequence-based prediction.

2.1. Performance comparison with existing methods

For evaluating the performance of the proposed model on the independent test dataset, we selected the ACP-DA and DeepACP.The independent test dataset ACP214 was retrieved from the combination of an alternate test dataset used in ACP-DA called ACP240 and

DeepACP with independent test datasets ACP162. However, to retrieve the sequence with lower redundancy, we applied CD-HIT of 0.35% over the concatenated dataset to remove the duplicate sequence from the test dataset. The performance comparison with those datasets with high redundancy 0.90% cut-off is not applicable, but to validate the performance of the model, we compared our proposed method performance with those control methods as shown in Table S1.

Table S1. Comparison of performance of existing method on independent test data and its performance metrics.

Dataset	Method	ACC%	MCC%
ACP240	ACP-DA	88.33	72.03
ACP162	DeepACP	82.93	66.32
ACP214*	ACP-ADA*	61.56	20.04

The reason for ACP-DA, and DeepACP to achieve the higher ACC and MCC was, most of the sequences are redundant in the test set. Those methods used the cut-off of 0.90% for training and independent test dataset and the number of sequences was higher in the proportion corresponding to positive and negative sequences for evaluation. However, we applied the peptide test set using CD-HIT of 0.35%, which has relatively fewer samples for evaluation in terms of ACC and MCC. Because of this, our method achieved an accuracy of 61.56% for the independent test dataset ACP214. Comparative to those methods, the data set we used for evaluation is different but achieved the remarkable ACC. Notation '*' represents the proposed method and newly constructed dataset used for the evaluation of model performance.

2.2. Training and Test Dataset with CD-HIT of 0.35%

All the training(ACP614) and test data(ACP214) were featured based on the sequential order information called Binary Profile Feature, Amino Acid Index, and Amino Acid Composition. Besides this, the evolutionary features called Position Specific Scoring Matrix(PSSM)[2] which have a robust prediction for the protein-related problem has been added to concatenated feature for evaluating the efficacy for recognition of anticancer peptides.

Motifs of evolutionary preserved peptide sequence provide important information relating to peptide and protein binding site and recognition. Different statistical predicting features can be extracted using these statistics. Mostly, Position Specific Scoring Metrics(PSSM) and Hidden Markov Model Features (HMM) were used for extracting the probability of the presence of certain amino acids at specific positions in the sequence to find similar protein sequences. For adding the evolutionary information with sequence order information, we choose PSSM as an additional feature to add with 210-dimensional sequential features. Though Hidden Markov features are gaining success in protein-related problems such as binding site prediction, we choose PSSM as an evolutionary feature and the dimension of the features is too large which may reduce the performance of the model. Machine learning models show weak performance for a high-dimensional feature for prediction.

PSSM features were extracted using PSI-BLAST v2.10.1(USA)[3].This method uses the UniRef90 database(v90,Washington DC,USA)[4]. The parameters and the setting of these both tools were configured as suggested by Zeng et al[5]. This methodology provided a 20-dimensional feature vector as output and normalized between 0 and 1 by using minimum and maximum values from overall features present in the training set.

2.3. Performance of machine learning classifiers on independent test data set without PSSM

We trained the machine learning models with this newly constructed dataset ACP614 and evaluated them on test data set following the similar configuration of redundancy

cutoff off of 0.35%. The performance of the models are visualized in the Table S2. In this method, the concatenated features [BPF+AAINDEX+AAC] were used. The prediction performance for the selected machine learning models was carried out.Comparing the results of different machine learning models, Adaptive boosting classifier performed well, for which the ACC and F1 scores were higher among all classifier. The adaptive boosting method(ADA) achieved on the test dataset an ACC of 64.32% and F1 score of MCC of 66.53%. Compared with the 5-fold cross validation method, using the data set with 0.90% cut off, the model performs poorly for the independent dataset ACP214 with CD-HIT of 0.35%. This result indicates that most of the sequences in the training samples were similar to the testing data set in ACP740 and ACP240, and ACP-DA,DeepACP,ACP-DL and AntiCP2.0 had achieved the higher ACC and F1 score value for the same reason.

Table S2. Performance of machine learning classifiers on independent test dataset ACP214 and its performance metrics(The best metrics are in bold)

Lx	Method	ACC%	PRE%	SEN%	SPE%	F1 Score%
50	MLP	57.47	62.34	59.64	55.04	61.32
50	SVM	57.09	60.32	68.34	44.32	63.32
50	RF	57.47	60.32	69.45	48.62	57.34
50	KNN	50.93	56.02	53.00	49.32	54.32
50	ET	56.02	59.03	68.03	42.03	63.12
50	GB	56.32	59.03	70.00	40.67	64.32
50	**ADA**	**64.32**	**63.42**	**69.98**	**58.00**	**66.53**

We evaluated the performance of the model selectively on the independent test dataset ACP214 with this length. Compared with other models such as MLP, SVM, RF, KN, ET, and GB, the ADA method achieved the maximum accuracy of 64.32% with F1 Score of 68.76%. This result suggests that ADA is capable of performing well on the dataset ACP614 and test dataset ACP214 as shown in Table S2.So, the ADA model is selected as the final classifier as it performed well on the test data set ACP214.

Comparing the performance of this model on the test dataset ACP214 is not reliable with the other control methods for classification of anticancer peptides because the test data we used are different and have cut-off threshold of 0.35%. All those control method used cut-off of 0.90% for both training and test datasets which is similar to our ACP740 and ACP240..

2.4. *Performance of machine learning classifiers on independent test dataset with PSSM*

In order to evaluate the efficacy of PSSM as a evolutionary sequence feature called as position specific scoring matrix, we concatenated this feature to the training dataset and test dataset created using the CD-HIT 0.35%.Furthermore we evaluated the performance of the machine learning model and compare the results on test dataset without PSSM and with PSSM as the additional feature.The robustness of sequence order feature and evolutionary feature for prediction is evaluated on our newly created test dataset ACP214 with CD-HIT 0.35%.In addition to the BPF, AAINDEX and AAC which resulted in 210 dimensional feature, for each sequence with the length of 50 were encoded into 50*20 dimensional features.The resulted dimension for each sequence was 50*20, where this dimension was flattened into 1000*1 to represent the each sequence.Hence the training and test data set sequence were represented with 1210 dimensional feature. Finally, to evaluate the performance of the model,we tested the efficacy of the model with PSSM as as sequential evolutionary features with sequence order feature hybridization and compared the model performance without PSSM and with PSSM interms of ACC and F1 Score.

Table S3. Performance of machine learning classifiers on independent test dataset ACP214 with PSSM (The best metrics are in bold)

Lx	Method	ACC%	PRE%	SEN%	SPE%	F1 Score%
50	MLP	54.05	49.04	73.08	39.00	59.02
50	SVM	54.05	49.03	71.03	40.13	54.54
50	RF	49.47	46.02	71.00	32.56	56.02
50	KNN	60.81	55.08	70.32	54.00	58.02
50	ET	54.72	49.56	74.01	39.00	59.00
50	GB	51.35	60.00	71.24	35.02	57.00
50	**ADA**	**62.42**	**61.42**	**64.42**	**58.00**	**62.32**

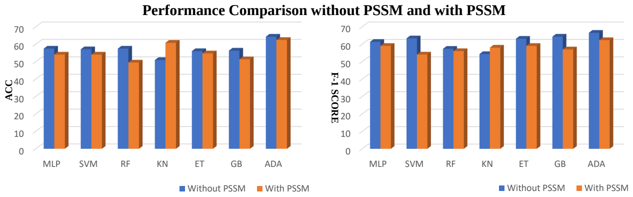

Figure S4. Comparison of model performance on test dataset ACP214 without PSSM and with PSSM.

We tested PSSM features on the training and test datasets created using the CD-HIT of 0.35%. The results show that the ADA model still performing the best in the prensence of PSSM features it is less in the case of training the model without PSSM features. The Table S3 shows the detailed results of different machine learning models on ACP214 using PSSM features. Figure S4 shows the comparison between different machine learning models on ACP214 with and without PSSM. The results suggest that including PSSM did not improve the performance of the final model.

3. Anticancer peptide recognition efficacy on length of peptide

We tested the lengths of 40, 50, and 60 and selected the length with best ACC. Experimentally, from 5-fold cross validation method on this different length of peptides with the two data sets: ACP740 and ACP240, most of the peptides with length >=50 were correctly classified as anticancer with high ACC and MCC values. On the other hand, the length 40 showed less performance as shown in Table S4.

Table S4. Performance of model on peptide with different length.

Dataset	Lx	ACC%	MCC%
ACP740	40	85.54	71.25
ACP740	**50**	**86.48**	**73.19**
ACP740	60	85.94	72.86
ACP240	40	86.66	73.19
ACP240	**50**	**90.83**	**81.65**
ACP240	60	90.78	78.30

References

1. Xue Ying,An Overview of Overfitting and its Solutions,*Journal of Physics: Conference Series* **2019**,1168
2. Sharzil Haris Khan, Hilal Tayara, Kil To Chong,ProB-Site: Protein Binding Site Prediction Using Local Features,*MDPI***2022**,*11*,2117
3. Stephen F. Altschul, Thomas L. Madden, Alejandro A. Schäffer, Jinghui Zhang, Zheng Zhang, Webb Miller, David J. Lipman:Gapped BLAST and PSI-BLAST: a new generation of protein database search programs ,*Nucleic Acids Research***1997**,*17*,3389-3402
4. Baris E. Suzek, Hongzhan Huang, Peter McGarvey, Raja Mazumder, Cathy H. Wu: UniRef: comprehensive and non-redundant UniProt reference clusterss,*Bioinformatics***2007**,*10*,1282-1288
5. Min Zeng, Fuhao Zhang, Fang-Xiang Wu, Yaohang Li, Jianxin Wang, Min Li,Protein–protein interaction site prediction through combining local and global features with deep neural networks,*Bioinformatics***2019**,*36*,1114-1120
