# Supplementary figures and images for: ACP-ADA: A Boosting Method with Data Augmentation for Improved Prediction of Anticancer Peptides
Source: Int J Mol Sci. 2022 Oct 13;23(20):12194. doi: 10.3390/ijms232012194 (PMC9603247; doi:10.3390/ijms232012194)

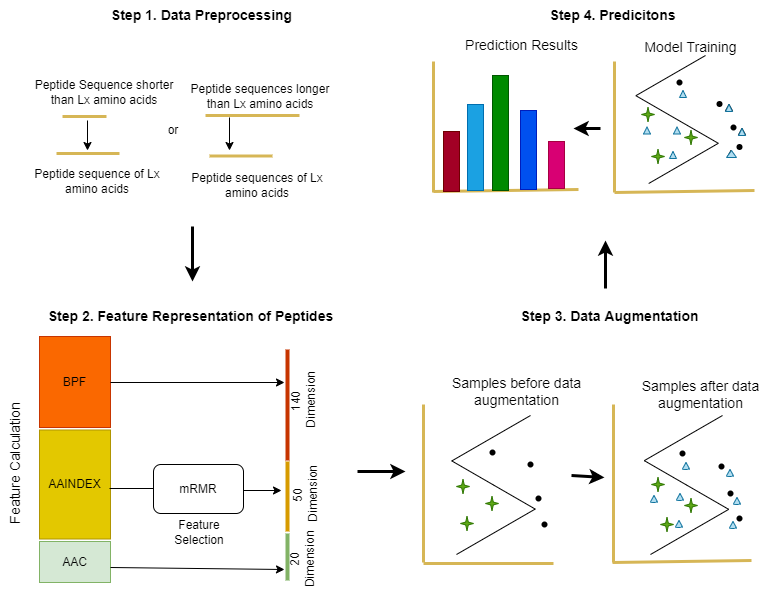

Supplement: Supplementary file 1 [file ijms-23-12194-s001.zip › Definitions/fig1.png]

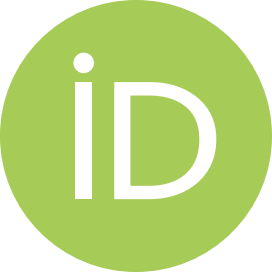

Supplement: Supplementary file 1 [file ijms-23-12194-s001.zip › Definitions/logo-orcid.pdf]

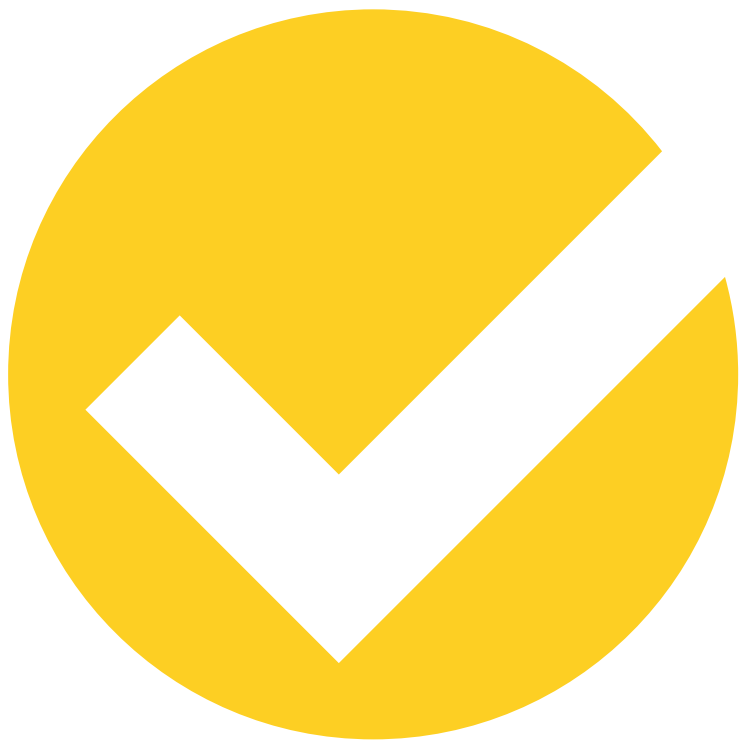

check for  
updates

Supplement: Supplementary file 1 [file ijms-23-12194-s001.zip › Definitions/logo-updates.pdf]

ACP740  
Feature Importance

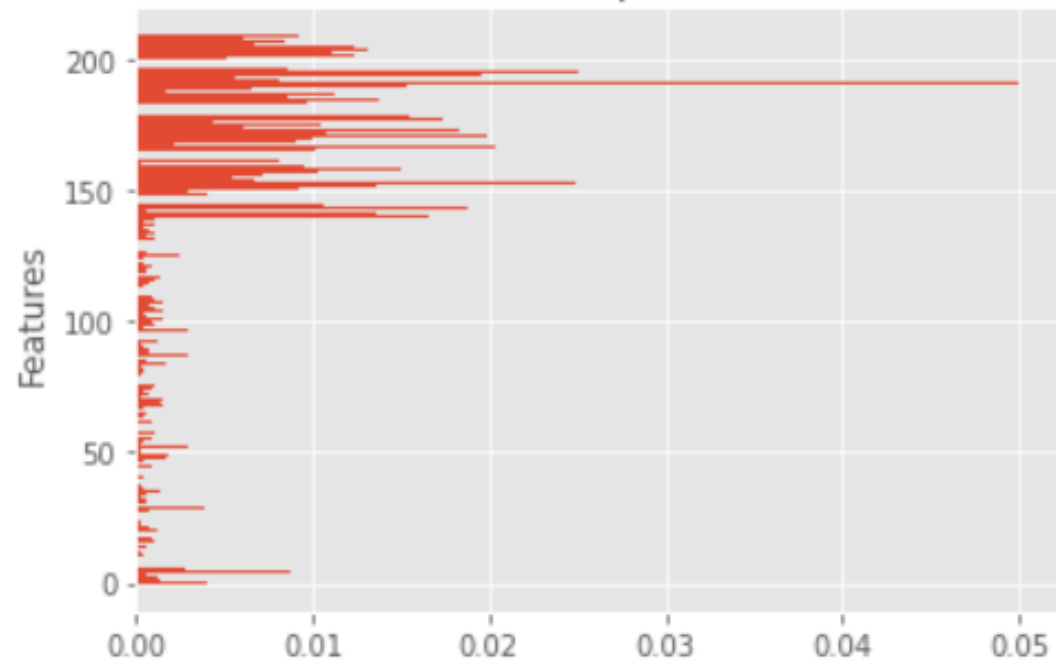

ACP240  
Feature Importance

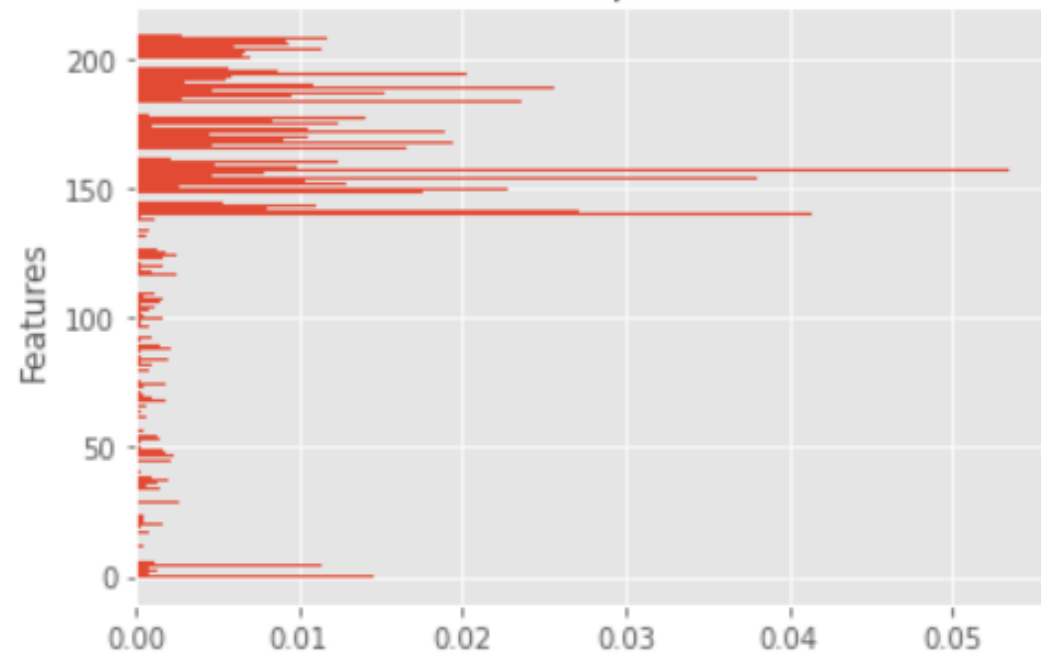

Supplement: Supplementary file 1 [file ijms-23-12194-s001.zip › feature.pdf]

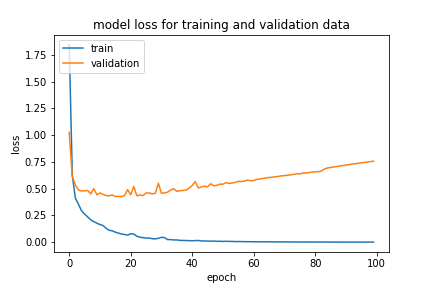

Supplement: Supplementary file 1 [file ijms-23-12194-s001.zip › loss_mlp.png]

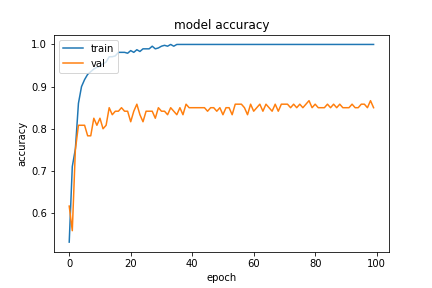

Supplement: Supplementary file 1 [file ijms-23-12194-s001.zip › mlp_accuracy.png]

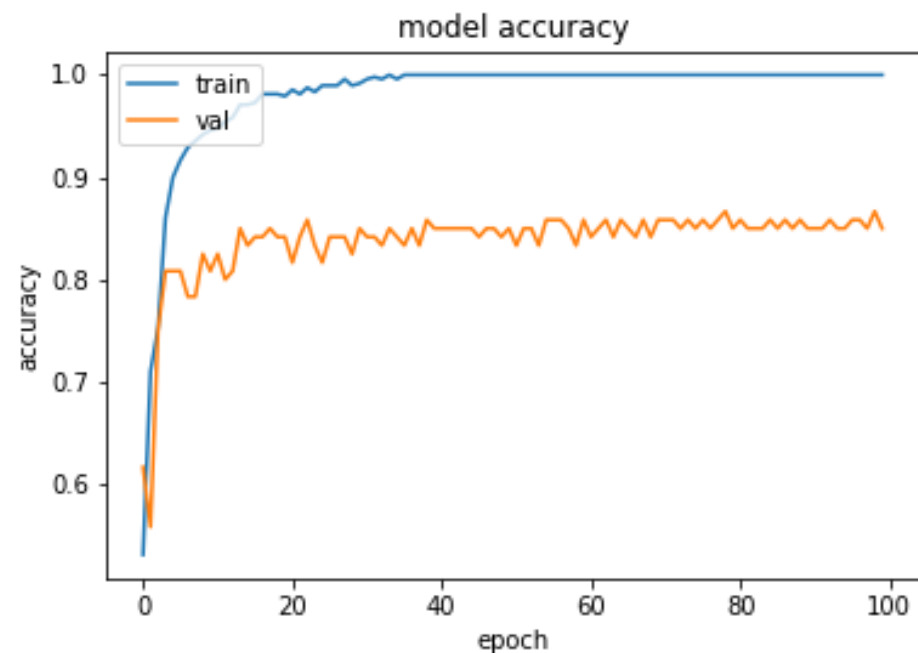

Overfitting problem in DNN

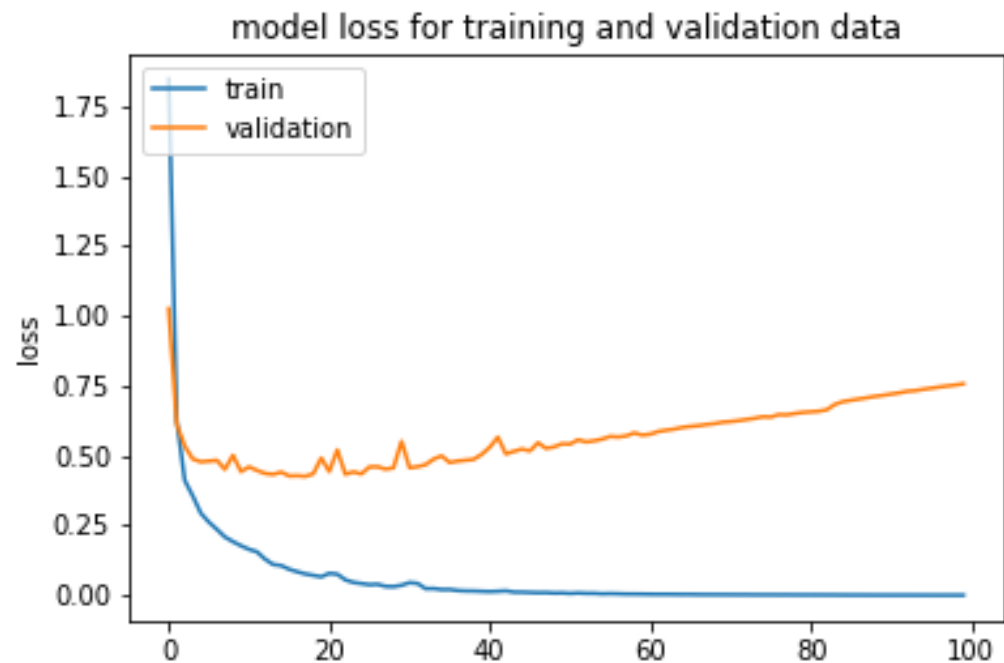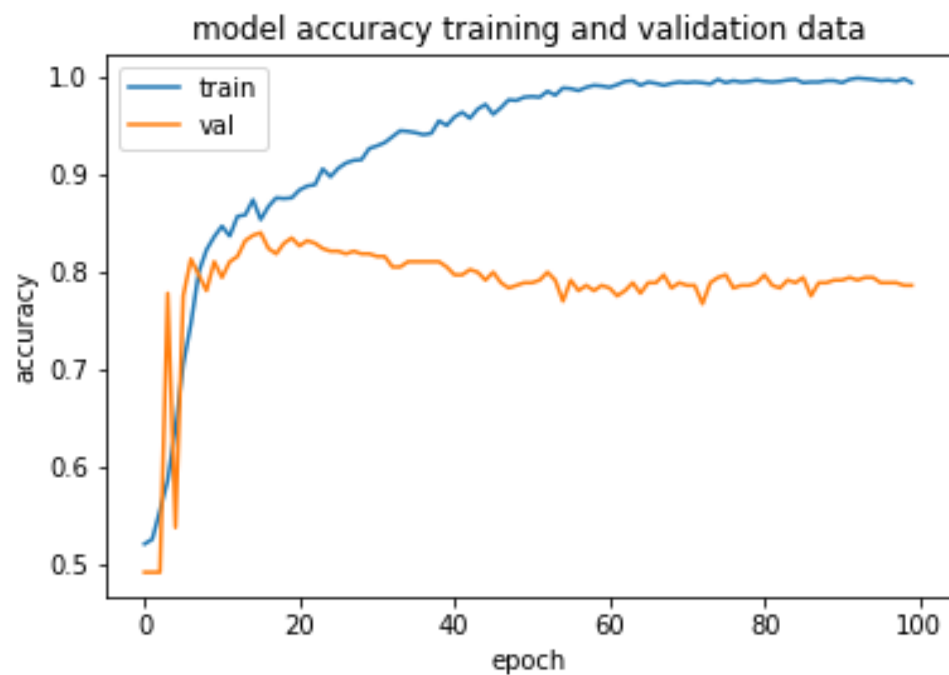

Overfitting problem in CNN

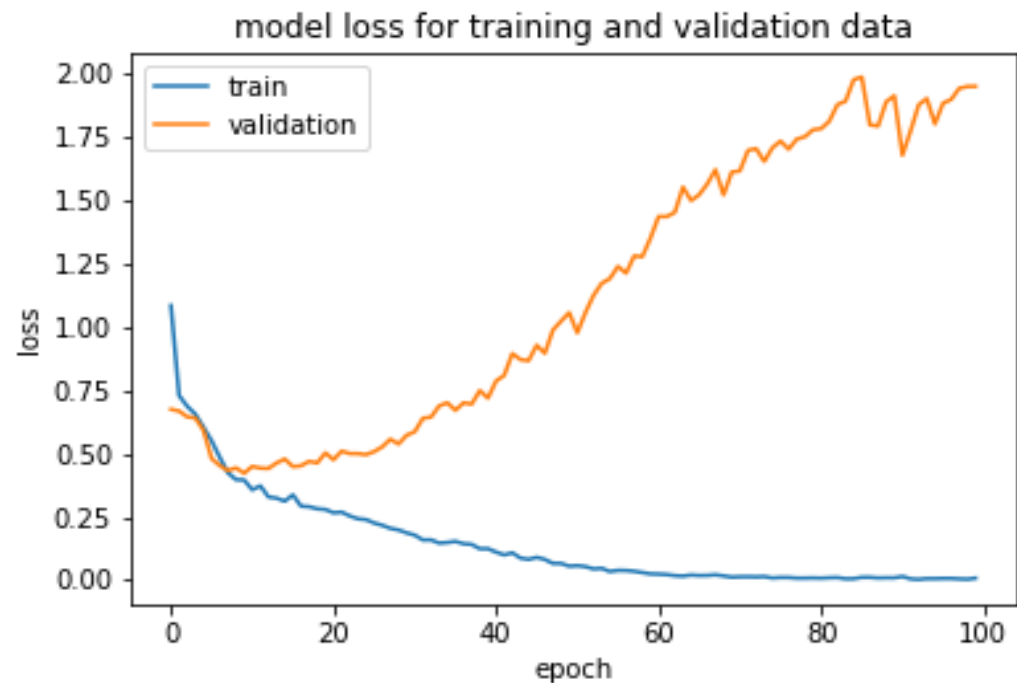

Supplement: Supplementary file 1 [file ijms-23-12194-s001.zip › OVERFITTING.pdf]

Performance Comparison without PSSM and with PSSM

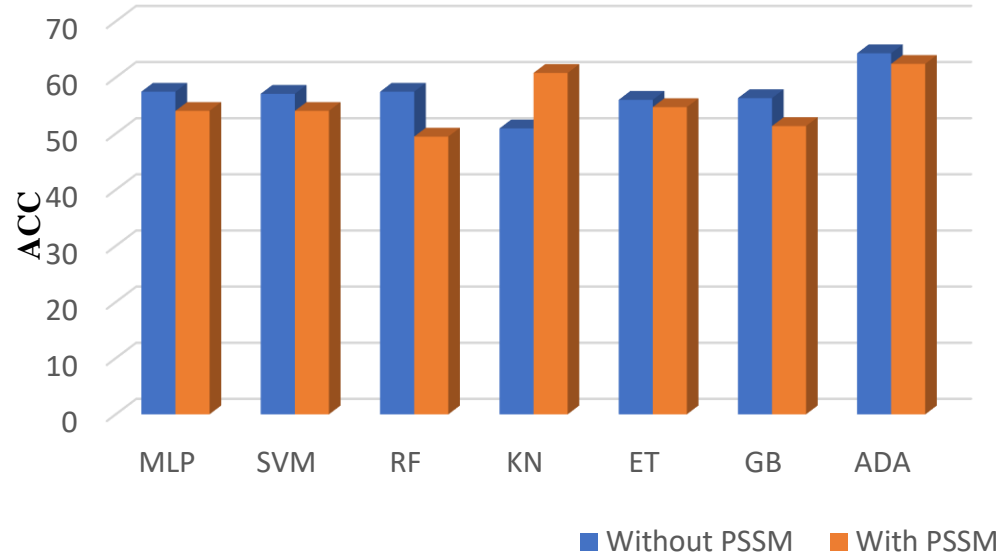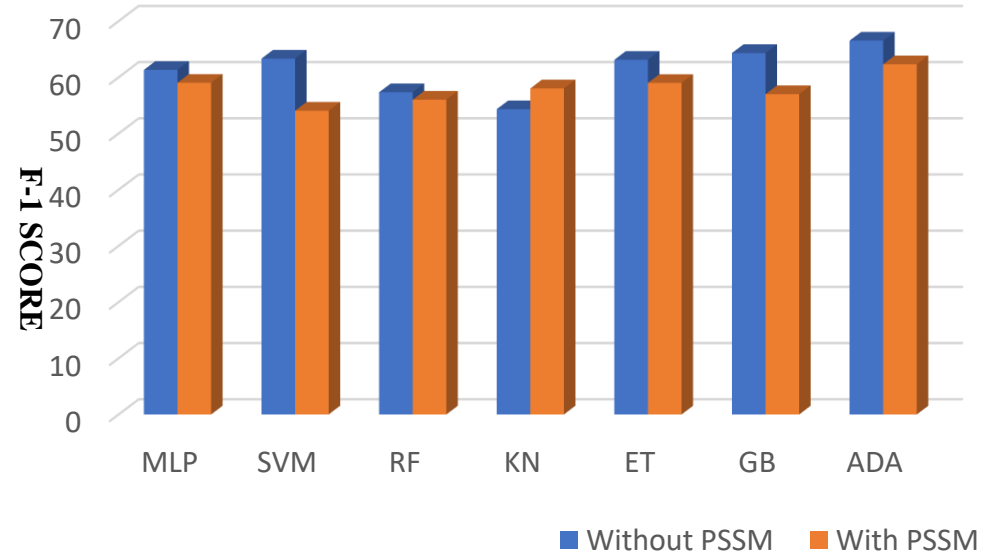

Supplement: Supplementary file 1 [file ijms-23-12194-s001.zip › pssm comparision.pdf]

Performance of Model on Independent Test Dataset-ACP214

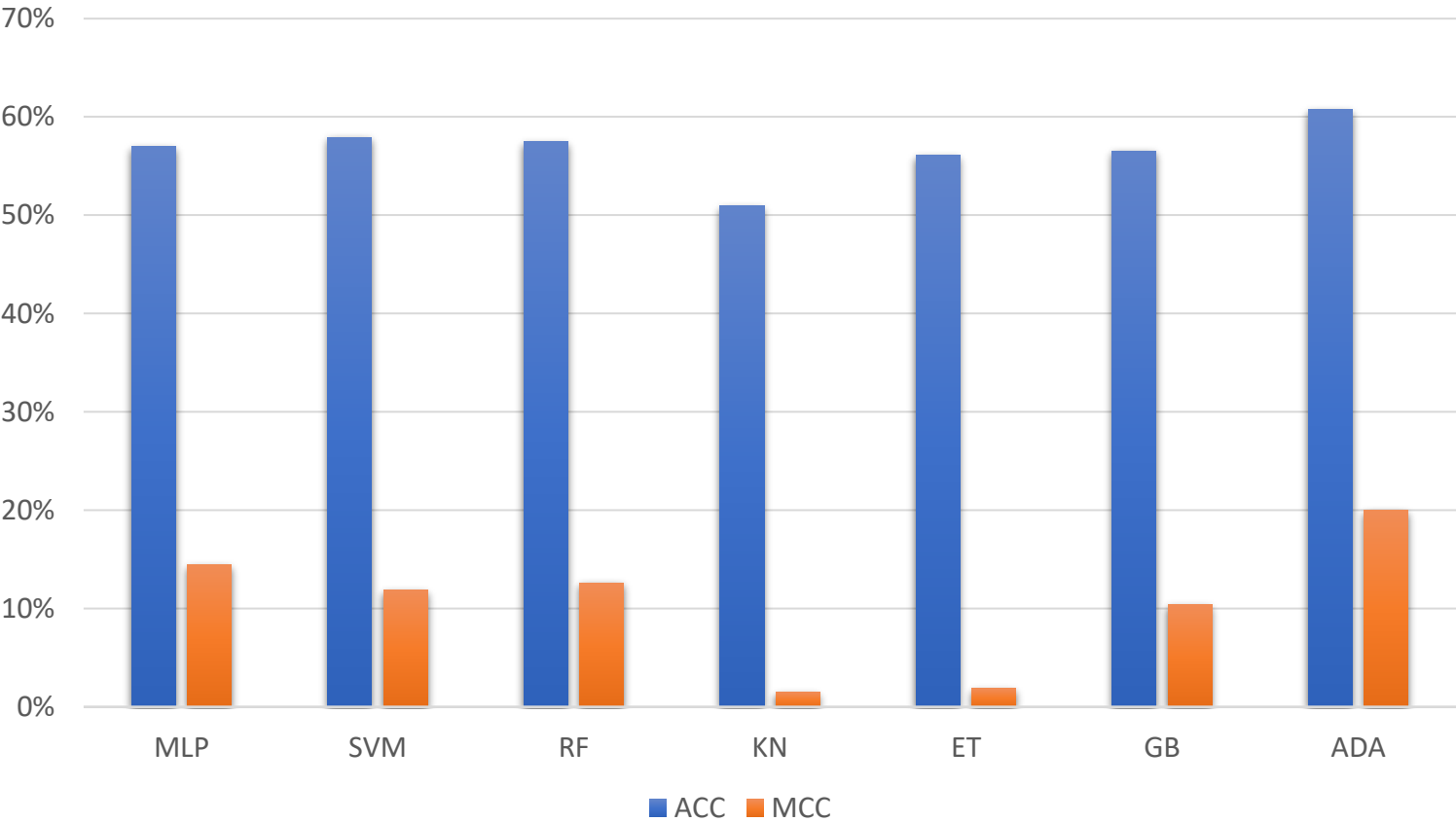

Supplement: Supplementary file 1 [file ijms-23-12194-s001.zip › SUPPFIG2.pdf]
